# Metagenomic analyses of a consortium for the bioremediation of hydrocarbons polluted soils

**DOI:** 10.1186/s13568-024-01764-7

**Published:** 2024-09-28

**Authors:** Emiliana Pandolfo, David Durán-Wendt, Ruben Martínez-Cuesta, Mónica Montoya, Laura Carrera-Ruiz, David Vazquez-Arias, Esther Blanco-Romero, Daniel Garrido-Sanz, Miguel Redondo-Nieto, Marta Martin, Rafael Rivilla

**Affiliations:** 1 Departamento de Biología, Facultad de Ciencias, Universidad Autónoma de Madrid, Darwin 2, 28049 Madrid, Spain; 2 Departamento de Química y Tecnología de Alimentos, Escuela Técnica Superior de Ingeniería Agronómica, Alimentaria y de Biosistemas, Universidad Politécnica de Madrid, Ciudad Universitaria, 28040 Madrid, Spain; 3 Department of Fundamental Microbiology, University of Lausanne, 1015 Lausanne, Switzerland

**Keywords:** Bacterial consortium, Bioremediation, Total petroleum hydrocarbons, Metagenomics, Metatranscriptomics

## Abstract

A bacterial consortium was isolated from a soil in Noblejas (Toledo, Spain) with a long history of mixed hydrocarbons pollution, by enrichment cultivation. Serial cultures of hydrocarbons polluted soil samples were grown in a minimal medium using diesel (1 mL/L) as the sole carbon and energy source. The bacterial composition of the Noblejas Consortium (NC) was determined by sequencing 16S rRNA gene amplicon libraries. The consortium contained around 50 amplicon sequence variants (ASVs) and the major populations belonged to the genera *Pseudomonas*, *Enterobacter*, *Delftia*, *Stenotrophomonas*, *Achromobacter*, *Acinetobacter*, *Novosphingobium*, *Allorhizobium*-*Neorhizobium*-*Rhizobium*, *Ochrobactrum* and *Luteibacter*. All other genera were below 1%. Metagenomic analysis of NC has shown a high abundance of genes encoding enzymes implicated in aliphatic and (poly) aromatic hydrocarbons degradation, and almost all pathways for hydrocarbon degradation are represented. Metagenomic analysis has also allowed the construction of metagenome assembled genomes (MAGs) for the major players of NC. Metatranscriptomic analysis has shown that several of the ASVs are implicated in hydrocarbon degradation, being *Pseudomonas*, *Acinetobacter* and *Delftia* the most active populations.

## Key points

- Bacterial consortium for hydrocarbon degradation developed by enrichment culture.
- Metagenomic analyses identified populations and genes for bioremediation.
- Metatranscriptomic analysis identified major actors and roles in bioremediation.

## Introduction

Total petroleum hydrocarbons (TPHs) are frequently polluting soils because of crude oil extraction, spills and accidents. Contamination by TPHs is a cause of concern and produce ecological damage. TPHs are a mixture of chemicals including aromatic hydrocarbons and aliphatic hydrocarbons composed of C5-C35 chains. Some of these chemicals are toxic, mutagenic and carcinogenic (Liu et al. 2011; Wang and Shao 2012). TPHs are complex mixtures with different structural configurations and a high degree of hydrophobicity. That makes TPHs hard to degrade by microorganisms and therefore they can be persistent in soils (Das and Chandran 2011). For these reasons, TPHs are considered priority pollutants by environmental protection agencies that have established standards for decontamination that range from 50 to 100 mg/kg in the USA. However the intervention value in several European countries has been set in 5000 mg/kg and 2000 mg/kg have been suggested as the maximum accepted TPH concentration for suitable soil management (Pinedo et al. 2013).

Bacteria are the major players in TPHs biodegradation and more than twenty-five bacterial genera containing hydrocarbonclastic bacteria have been identified (Das and Chandran 2011). Other, more recent studies have found that bacteria from, at least, 80 genera are able to degrade TPHs (Tremblay et al. 2017). Several of these bacteria including (in alphabetical order) *Achromobacter*, *Acinetobacter*, *Alkanindiges*, *Alteromonas*, *Arthrobacter*, *Burkholderia*, *Dietzia*, *Enterobacter*, *Kocuria*, *Marinobacter*, *Mycobacterium*, *Pandoraea*, *Pseudomonas*, *Rhodococcus*, *Staphylococcus*, *Streptobacillus* and *Streptococcus* have been described to play important roles in TPHs biodegradation (Margesin et al. 2003; Chaerun et al. 2004; Jin et al. 2012; Nie et al. 2014; Varjani and Upasani 2016; Sarkar et al. 2017; Varjani 2017; Xu et al. 2017). Bacteria degrade aliphatic hydrocarbons aerobically through any of four different pathways: terminal carbon oxidation (Li et al. 2008), subterminal oxidation (Watkinson and Morgan 1990), biterminal oxidation (Watkinson and Morgan 1990) and alkane dioxidation (Maeng et al. 1996). All these pathways converge in beta-oxidation (Watkinson and Morgan 1990). The starting enzymes in these pathways are typically alkane monooxigenases, belonging to different families (Van Beilen et al. 2003). The bacterial aerobic degradation of aromatic and polyaromatic compounds is based in many peripheral pathways that converge in a few intermediaries, such as cathecol and protochatecuate (Revised in (Ghosal et al. 2016)). In these intermediaries, the aromatic ring is broken by dioxygenases that can cleave in meta or orto, finally yielding tricarboxylic acid pathway intermediaries (Cerniglia 1993).

However, TPHs are complex mixtures of hydrocarbons with a high degree of heterogeneity. Therefore, there are no single bacterial strains able to fully degrade all hydrocarbons (Al-Sayegh et al. 2015; Xu et al. 2018). This has led to the development of bacterial consortia that can cooperatively degrade these complex mixtures (Garrido-Sanz et al. 2018, 2019; Wu et al. 2017). These consortia can therefore be used as inoculants in soil bioremediation technologies involving bioaugmentation, such as Biopiles (Azubuike et al. 2016) and Ecopiles (Martínez-Cuesta et al. 2023).

Metagenomics have been proved to be useful in determining the bacterial populations in a consortium, identifying the key genes implicated in biodegradation and establishing the specific roles of each of the bacterial populations present in the consortium (Garrido-Sanz et al. 2019). However, metagenomic analysis is not sufficient to determine the most active populations in the biodegradation process. Here, we have included metatranscriptomics for this purpose. Therefore, the aim of this study was to generate a natural consortium able to degrade TPHs and to determine whether a combination of metagenomic and metatranscriptomic analyses was enough to fully characterize it, without bacterial isolation and biochemical determination of hydrocarbon degradation.

For this purpose, we have used an enrichment culture from a soil polluted with TPHs to generate a TPHs degradation consortium. We have used diesel as the sole carbon and energy source as a proxy for TPHs. Diesel is a complex mixture of hydrocarbon molecules consisting of approximately 75% aliphatic hydrocarbons and about 25% aromatic hydrocarbons, mostly containing between 9 and 25 carbon atoms (Olonoff 2014). The results reported here show that a combination of metagenomic and metatranscriptomic analyses can characterize the bacterial populations present in a bioremediation consortium, their possible role in the biodegradation processes and can identify the most active populations in biodegradation.

## Materials and methods

### Isolation of the bacterial consortium and DNA and RNA extraction

The consortium was isolated from soil from a machinery park area in Noblejas (Toledo, Spain. 39.96865, -3.41455), contaminated with different hydrocarbons, mineral oils, and heavy metals. Total petroleum hydrocarbons (TPHs) concentration was 4,051 mg/kg (wet soil) and heavy metals concentrations (mg/kg) were Arsenic 77.3; Cadmium 7.8; Chrome 14.9; Copper 8.5; Nickel 9.9; Lead 339.2; Zinc 680.5 (Curiel-Alegre et al. 2022). Soil and consortium described here are the same used in (Curiel-Alegre et al. 2022).

A soil sample consisting of three replicates (named R1, R2 and R3) was collected and sieved. A sequential enrichment culture procedure was used to isolate the diesel-degrading bacterial consortium. The method used to isolate consortia and extract DNA in this work is the same as that used by Garrido-Sanz et al. (2019). Briefly, 2 g (wet weight) of diesel-polluted soil was added to 500 mL of sterile minimum salt medium (MM), that was supplemented with 1 mL/L of phosphate-buffered mineral medium salts (PAS) and 0.005% of yeast extract (Garrido-Sanz et al. 2018), and grown at 28 °C with shaking (140 g) in Erlenmeyer flasks for 48 h. One mL/L of diesel was added as the sole carbon and energy source. After five 48 h consecutive subcultures, the control subculture without carbon and energy sources showed no growth, demonstrating that growth in the consortium was due to the use of diesel as the sole carbon and energy source. At this time, 20 mL aliquots of the 48 h enrichment culture were centrifugated for 10 min at 4000 g and the pellets resuspended in glycerol 80% and deep- frozen at − 80 °C, generating the isolated consortia (Noblejas Consortium). DNA extraction from the bacterial consortium after 48 h of growth from a glycerol stock was performed using the Realpure Genomic DNA Extraction Kit (Durviz, Spain). Bacterial consortium DNA concentration was determined using Qubit fluorometer (Thermo Fisher Scientific Inc).

For RNA extraction, the consortium was grown from a glycerol stock in the previous described media and conditions. RNA was isolated from three independent cultures after 72 h of growth following the protocol described by Blanco-Romero et al. (2022). Briefly, the bacterial cultures were centrifuged for 15 min at 4200 g. Then, supernatant was discarded, and the pellet resuspended in 1 mL NaCl 0.85% (w/v), centrifuged for 1 min at 8000 g, frozen with liquid nitrogen and stored at − 80 °C. Frozen pellets were resuspended in 200 µL of lysis buffer (Tris-HCl 1.0 M, EDTA 0.1 M pH 8.0, and 0.4 mg/mL lysozyme) and incubated for 5 min at Room Temperature (RT). Total RNA was extracted using the TRIzol-chloroform method. Briefly, 1 mL of TRIzol was added to each sample and incubated for 5 min at RT. Subsequently, 0.2 mL of chloroform was added, and incubated for 3 min at RT. Samples were centrifuged for 15 min at 12,000 g. The aqueous phase was transferred to a clean tube and 0.5 mL of isopropanol added. Samples were then agitated by inversion and kept for 10 min and further centrifuged for 10 min at 12,000 g. The supernatant was discarded, and the RNA pellet was cleaned with 1 mL of 75% (v/v) ethanol. Cleaned samples were centrifuged 5 min at 7,500 g and 2 min at 7500 g to assure complete ethanol removal. Samples were air-dried at RT for 5 min and resuspended in 20 µL of RNase-free water for 5 min at 4 °C. Subsequently, 1 µg of RNA was treated with DNase I (RQ1 Promega) following the instructions provided by the manufacturer and RNA Clean and Concentrator kit (Zymo Research), and finally resuspended in 15 µL of RNase-free water. 1 U/µL of SUPERase-InTM RNase inhibitor (ThermoFisher Scientific) was added before storing the samples at − 80 °C. Bacterial consortium RNA concentration was estimated using Qubit fluorometer (Thermo Fisher Scientific Inc).

### Microbiome analysis

For the profiling of the bacterial community, the isolated DNA underwent sequencing of the V3-V4 16S rRNA amplicons at the Genomic Services facility located at the Parque Científico de Madrid (Spain). The sequencing employed the primers 341F (5’-CCT ACG GGN GGC WGC AG-3’) and 805R (5’-GAC TAC HVG GGT ATC TAA TCC-3’) (Herlemann et al. 2011). Briefly, libraries were prepared in accordance with Illumina MiSeq v3 reagent kit specifications and subjected to sequencing on the Illumina MiSeq System, producing 2 × 300 bp reads. Preprocessing involved the removal of read regions corresponding to the oligonucleotides used in the amplification, as well as any residual adapter traces, using Cutadapt software (Martin 2011). Reads denoising and clustering for inference of amplicon sequence variants (ASVs) was carried out using the R package DADA2 v1.18 (Callahan et al. 2016). ASVs abundance per sample and their corresponding nucleotide sequences were loaded into the QIIME2 v2-2021.2 platform (Bolyen et al. 2019), to infer phylogenetic distances and taxonomic assignment of ASVs. Briefly, the QIIME2 phylogeny plugin and its align-to-tree-mafft-fastree pipeline (Katoh and Standley 2013; Price et al. 2010) were used to align ASVs sequences and calculate a rooted phylogenetic tree. Taxonomic assignment of ASVs was performed using the SILVA 99% SSU rRNA sequence database release 138 (Quast et al. 2013). Sequences in the database were trimmed to include only the V3-V4 amplicon region used in this study and a naive Bayes classifier (Murphy 2006) was then constructed ASVs comparison against the classifier was done using the QIIME2 classify.sklearn tool in feature-classifier plugin (Pedregosa et al. 2011). Taxonomic assignation tables and phylogenetic tree were imported into R using qiime2R package (Bisanz 2018; Bokulich et al. 2018) and were combined with the ASVs abundance table to build a Phyloseq object (McMurdie and Holmes 2013) to prepare graphical representation and diversity studies of the communities.

### Whole metagenome and metatranscriptome shotgun sequencing and analysis

For metagenomic analysis, total DNA extracted from the microbial consortium underwent custom sequencing at Parque Científico de Madrid (Madrid, Spain) using the Illumina MiSeq 2 × 300 system. The shotgun library preparation was performed utilizing the TruSeq kit. For metatranscriptomic analysis, RNA samples were sequenced in triplicate using the Illumina NextSeq 2 × 150 system at a depth of approximately 2.5 Gb per sample by Sistemas Genómicos (Madrid, Spain). For both, sequencing and library preparation procedures were executed by the sequencing facilities.

Trimmomatic software v0.39 (Bolger et al. 2014) was used to remove Illumina adapters from raw reads, low quality reads and those smaller than 100 nts. Filtered and trimmed paired reads (96% of the original reads were recovered for the metagenome and 98% for the metatranscriptome) were used as input for the SqueezeMeta v1.1.0 (Tamames and Puente-Sánchez 2019) pipeline for assembly and functional annotation. Metagenomic data reads were utilized for the assembly process, and contigs with lengths exceeding 500 base pairs were retained, while metatranscriptomic reads were mapped onto the generated assembly, ensuring a comprehensive and integrated analysis of genomic binning, taxonomic composition, and transcriptomic information. The abundance of genes of interest in the consortium was quantified using the Transcripts per Million (TPM) unit, though commonly used for gene expression normalization, can also be used in metagenomic and metatranscriptomic studies to study functional pathways. Additionally, to corroborate the taxonomic assignment for each bin, fasta files from each bin were submitted to TYGS (Type-Strain Genome Server) web to get the correspondence between the genomic sequence and a bacterial species.

The results were imported and analyzed in R using the SQMtools package (Puente-Sanchez et al. 2020), as well as the readr (Wickham 2023), tibble (Müller 2023), dplyr (Wickham 2023b), tidyr (Wickham, Vaughan, and Girlich 2023), ggplot2 (Wickham 2016), patchwork (Pedersen 2023) and Pheatmap (Kolde 2023) packages for data table processing and plots creation.

The functional analysis of TPHs degradation focused on assessing the presence and relative abundance of thirty-five selected genes implicated in the initial steps of hydrocarbon biodegradation within the metagenome. The details of the tested genes are presented in Table 1. These genes were selected because they encode the known initial enzymes of both peripheric and central pathways for the degradation of aliphatic and aromatic hydrocarbons. Pathways and enzyme functions are indicated by the KEGG identity of the enzymes encoded by the selected genes.

**Table 1:** Compilation of chosen hydrocarbon pathways and associated genes with their respective KEGG codes utilized in this study

Hydrocarbon	Metabolic pathway	Gene Name	KEGG ID
Aromatic	Peripheral pathway	*bphAa*	K08689
		*bphC*	K00462
		*benA*	K05549
		*pobA*	K00481
		*carAa*	K15751
		*antA*	K05599
		*nahAa*	K14581
		*nahC*	K14583
		*nahAc*	K14579
		*nagG*	K18242
		*poxA*	K16249
		*etbAa*	K14748
		*nidA*	K11943
		*phdF*	K11945
		*pht3*	K18068
		*phtAa*	K18251
		*tphA2*	K18074
		*cmtAb*	K10619
		*cmtC*	K10621
		*hcaE*	K05708
		*mhpB*	K05713
	Central pathway	*catA*	K03381
		*catE*	K07104
		*pcaG*	K00448
		*ligA*	K04100
		*hmgA*	K00451
		*chqB*	K04098
		*badA*	K04110
		*boxA*	K15511
		*boxB*	K15512
		*boxC*	K15513
		*boxD*	K15514
Aliphatic	Subterminal oxidation pathway	*acmB*	K18372
	Terminal oxidation pathway	*alkB*	K00496
		*ladA*	K20938

## Results

### Microbiome analysis based on 16S rRNA gene amplicon libraries

DNA extracted from the consortium derived from Noblejas soils was used to prepare 16S amplicons libraries that were sequenced and analyzed through QIIME2. The rarefaction curve for the consortium was obtained showing that the Noblejas consortium (NC) contains ca. 50 ASVs. The curve was saturated, and a full community coverage was achieved before 50,000 sequences, so all biodiversity has been analyzed and the presence of other taxa in the consortium is unlikely.

**Fig. 1:**
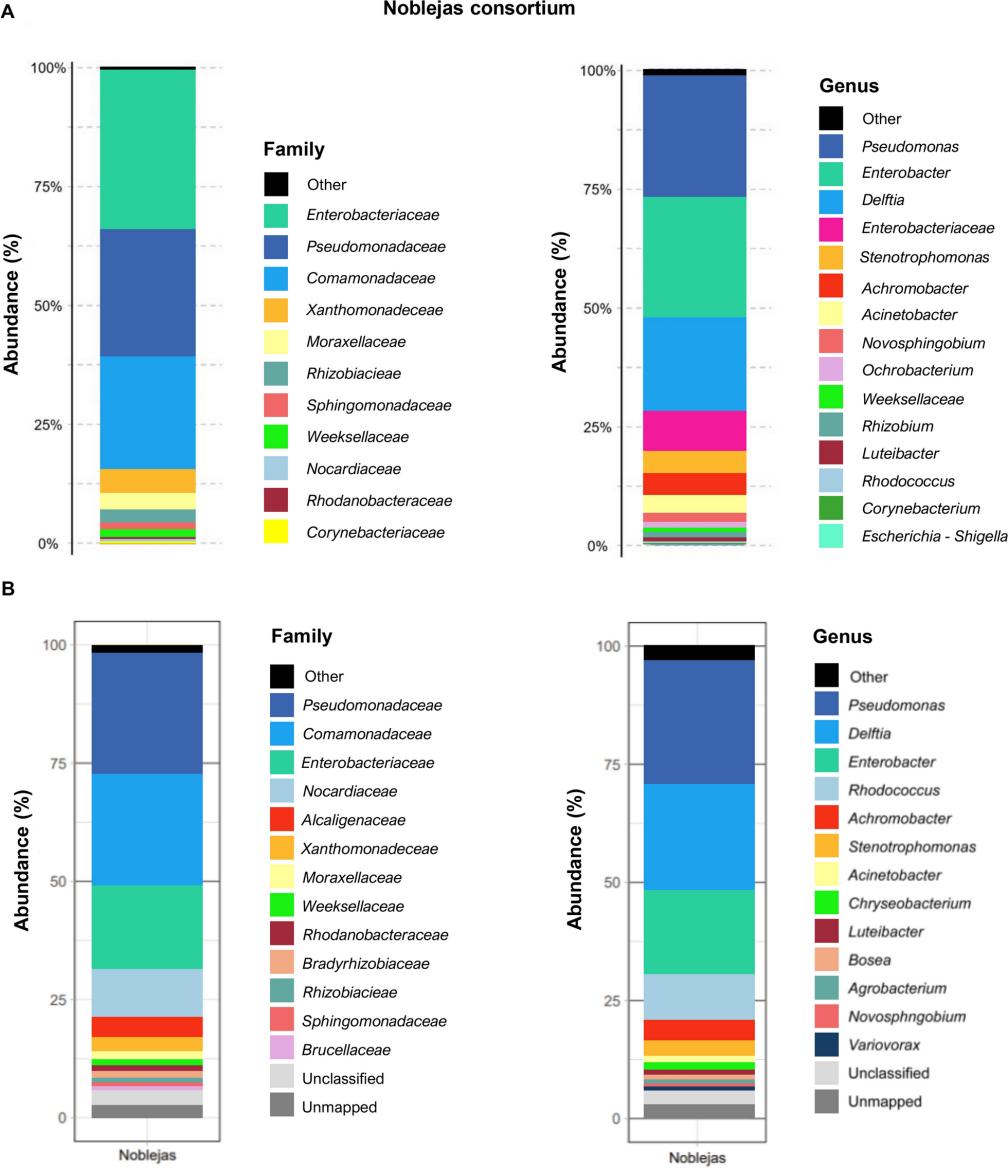
Taxonomic assignment. **A** Relative abundance of bacteria from Noblejas consortia at the level of families and genera based on 16S rRNA taxonomic assignment. **B** Relative abundance of bacterial based on whole metagenome shotgun analysis. Families and genera with a relative abundance lower than 0,5% are grouped under the “other” category. Unmapped refers to the proportion of reads that could not be assigned to any contig. Unclassified points to those contigs that could not be assigned to any Taxa

At the Phylum level (not shown), the consortium is dominated by *Pseudomonadota* (98.25%). Other significant phyla are *Actinomycetota* and *Bacteroidota*. Figure 1A shows the composition of the bacterial community of the consortium at family level. The composition of Noblejas consortium is dominated by *Enterobacteriaceae* (33.56%), followed by *Pseudomonadaceae* (26.61%) *Comamonadaceae* (24.35%), *Moraxellaceae* (3.64%), *Xantomonadaceae* (4.40%), *Rhizobiaceae* (2.42%), *Sphingomonadaceae* (1.87%) and *Rhodanobacteriaceae* (1%). All other families are below 1%. At the genus level (Fig. 1A), the Noblejas consortium is formed by *Pseudomonas* (26.61%), *Enterobacter* (24.96%), *Delftia* (20.14%), other *Enterobacteriaceae* (8.61%), *Stenotrophomonas* (4.40%), *Achromobacter* (3.80%), *Acinetobacter* (3.64%), *Novosphingobium* (1.81%), *Allorhizobium*-*Neorhizobium*-*Rhizobium* (1.35%), *Ochrobactrum* (1.04%) and *Luteibacter* (1.00%). All other genera are below 1%.

### Microbiome analysis based in metagenomics DNA analysis

DNA isolated from the consortium was also used for Whole Metagenome Sequencing (WMS) and microbial diversity was estimated by using the phylogenomic assignment of coding sequences (CDS). Comparing the taxa bar plots of the taxonomic assignment made by using 16S rRNA genes sequences (microbiome analysis of the consortia) with the ones made by using CDSs (metagenome analysis of the consortia), we observed that the same principal families and genera are present. At the family level, the Noblejas consortium (Fig. 1B) presents a community formed by *Pseudomonadaceae* (26.7%), *Comamonadaceae* (25.05%), *Enterobacteriaceae* (18.52%), *Nocardiaceae* (10.27%), *Alcaligenaceae* (4.77%), *Xanthomonadaceae* (3.12%), *Moraxellaceae* (1.48%), *Weeksellaceae* (1.45%), *Rhodanobacteriaceae* (1.42%), *Rhizobiaceae* (1.04%) and *Sphingomonadaceae* (1.0%). At genus level (Fig. 1B) the prevalent taxa are *Pseudomonas* (26.7%), *Delftia* (23.44%), *Enterobacter* (18.31%), *Rhodococcus* (10.21%), *Achromobacter* (4.73%), *Stenotrophomonas* (3.12%), *Acinetobacter* (1.48%) and *Chyrseobacterium* (1.45%). All other families and genera are below 1%.

### Metagenome assembled genomes (MAGs)

From the WMS data, through binning using the Squeeze-Meta pipeline, it has been possible to reconstruct 12 MAGs from the Noblejas consortium (Table 2) which corresponded with the most abundant ASVs identified in the consortium. Their genomic sizes and %GC content are in good agreement with the closest relative genome. Eight of these MAGs show more than 90% completeness. Seven of the MAGs were identified to the species level (dDDH > 70).

**Table 2:** Genomic statistics of the MAGs reconstructed from the whole metagenomic sequence of the Noblejas consortium

SqueezeMeta Taxonomic Assignation	Closest relative genome (TYGS)	Completeness	dDDH (%)	Length (bp)	GC (%)	Contigs
*Acinetobacter*	*Acinetobacter calcoaceticus*	99.26	62.5	3,592,269	38.62	72
*Pseudomonas*	*Pseudomonas vlassakiae*	98.86	44.0	5,767,948	61.85	55
*Rhodococcus*	*Rhodococcus jialingiae*	98.72	86.1	7,579,905	62.37	743
*Stenotrophomonas*	*Stenotrophomonas geniculata*	98.02	82.9	4,373,997	66.60	43
*Gammaproteobacteria*	*Enterobacter ludwigii*	97.72	92.7	4,621,498	54.66	37
*Chryseobacterium*	*Chryseobacterium hispalense*	97.46	72.6	4,133,185	36.56	62
*Pseudomonas*	*Pseudomonas frederiksbergensis*	93.77	78.9	7,126,799	58.55	544
*Pseudomonas*	*Pseudomonas rhizophila*	92.16	63.0	8,414,776	61.03	1272
*Proteobacteria*	*Luteibacter flocculans*	88.47	54.0	3,863,551	65.28	359
*Delftia*	*Delftia acidovorans*	83.58	84.4	6,468,209	66.82	30
*Agrobacterium*	*Agrobacterium radiobacter*	83.39	31.9	4,025,303	59.77	597
*Bosea*	*Bosea robiniae*	77.90	70.4	4,904,137	66.13	773

### Genes for metabolism of hydrocarbon compounds present in the consortium

**Fig. 2:**
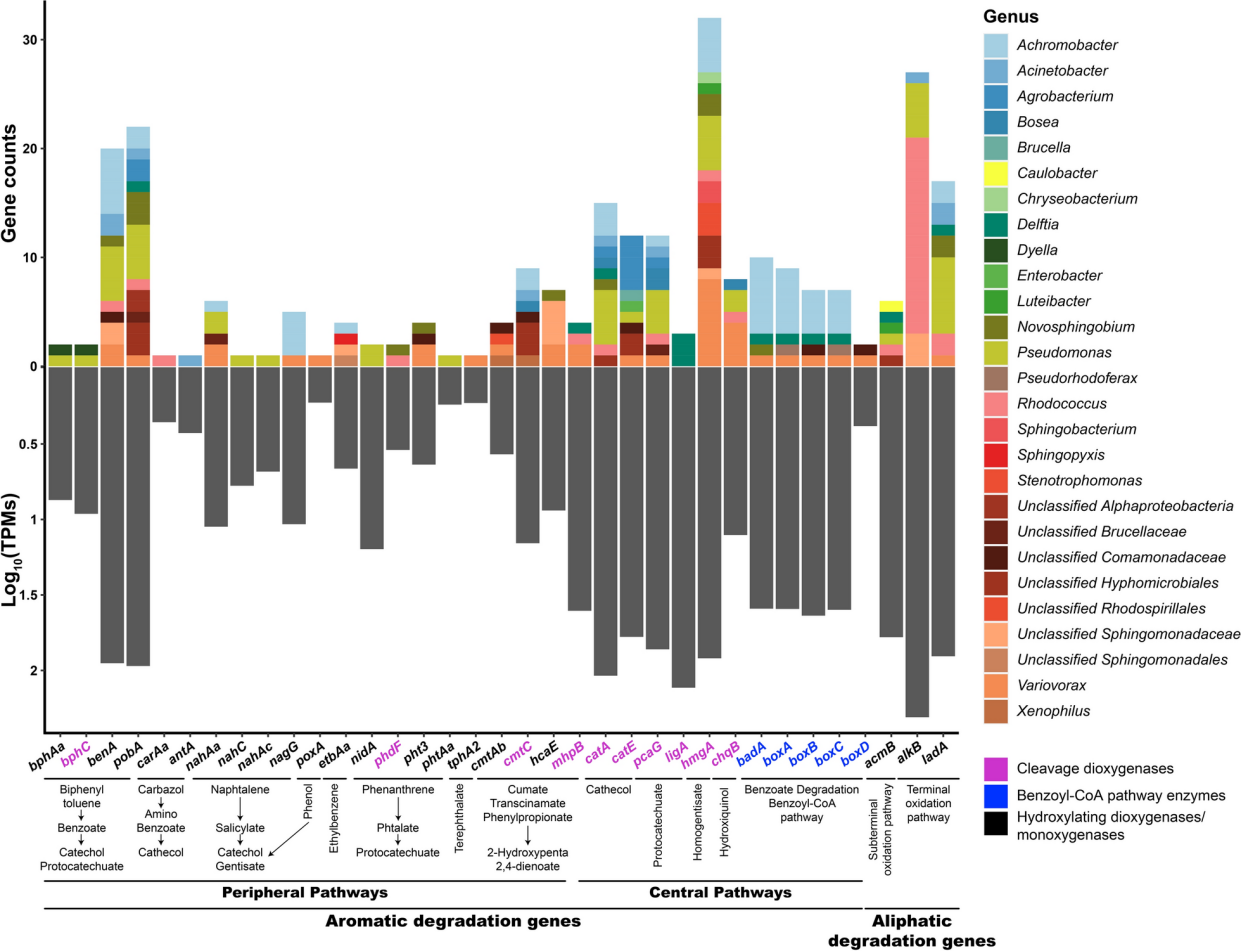
Presence and abundance, in terms of number of genes and TPM values, of genes that encode enzymes that are involved in pathways for the degradation of aliphatic and aromatic hydrocarbons and were identified in the metagenome and classified at the genus level in the Noblejas consortium. Abscissa axe labels are grouped according to metabolic pathways and colors indicate enzymatic activity class

Whole metagenomic sequencing also allowed for the identification of genes encoding relevant enzymes implicated in hydrocarbons biodegradation. The results of the identification of genes encoding alkane-degrading enzymes in the metagenome of the Noblejas consortium are summarized in Fig. 2.

All the 35 searched genes were found in the consortium, indicating that all pathways implicated in aliphatic, aromatic and polyaromatic pathways that were searched for, were present in the consortium. Genes implicated in aliphatic hydrocarbon degradation are abundant and widespread in the consortium. AlkB (alkane 1- monooxygenase) is a non-heme iron membrane protein that carries out the hydroxylation of the terminal carbon of C5-C12 n-alkanes, oxidizing them to 1-alkanols (Van Beilen et al. 2003). In the Noblejas consortium, *alkB* gene copies belong mostly to *Rhodococcus* (eighteen copies), *Pseudomonas* (five copies), to *Sphingomonadaceae* (three copies) and one copy to the *Acinetobacter* genus. LadA (long-chain alkane monooxygenase) also participates in the terminal oxidation pathway of n-alkanes. LadA is a monooxygenase that catalyzes the oxidation of the terminal carbon in long-chain alkanes (Ji et al. 2013; Li et al. 2008). In the Noblejas consortium there are seventeen copies of *ladA* mostly corresponding to *Pseudomonas* (seven copies) *Acinetobacter*, *Achromobacter*, *Rhodococcus* and *Novospingobium* (two copies each) and *Delftia* and *Variovorax* (one copy each). Genes encoding enzymes involved in subterminal alkane degradation are represented by *acmB*. Several copies of this gene have been found, corresponding to *Rhodococcus*, *Pseudomonas*, *Luteibacter*, *Delftia* and *Caulobacter*.

Genes that encode enzymes implicated in the degradation pathways of aromatic hydrocarbons and identified within the metagenome of the Noblejas consortium are also shown in Fig. 2. Biphenyl 2,3-dioxygenase (encoded by *bphAa*) and dihydroxybiphenyl 2,3-dioxygenase (encoded by *bphC*) were found in the Noblejas consortium with genes copies assigned to the *Pseudomonas* and *Dyella* genera, suggesting that the Noblejas consortium has the ability to degrade biphenyl and co-metabolize PCBs. Benzoate 1,2-dioxygenase (encoded by *benA*) (Neidle et al. 1991) is an enzyme involved in oxidation of the benzoate ring. In the Noblejas consortium, as many as twenty-one copies of the gene were found. Most of the copies belonged to *Pseudomonas* and *Achromobacter* (five and six copies respectively), followed by an unidentified *Sphingomonadaceae*, *Variovorax* and *Acinetobacter* (two copies each), and *Novosphingobium*, *Rhodococcus* and an unknown genus of *Comomaonadaceae* family (one copy each).

Hydroxybenzoate 3-monooxygenase (encoded by the *pobA* gene) (Entsch et al. 1988) was found with twenty-two copies in the Noblejas consortium. *Pseudomonas* had five copies followed by *Novosphingobium* and non-classified *Hyphomicrobiales* (three copies each) while *Agrobacterium*, *Achromobacter* and an unclassified *Alphaproteobacteria* had two copies each. One gene copy was assigned to *Delftia*, *Acinetobacter*, *Rhodococcus*, *Variovorax* and to an unclassified *Brucellaceae*. Carbazole 1,9a-dioxygenase (encoded by *carAa* gene) catalyses the first reaction in the pathway of carbazole degradation. It was only found with one copy assigned to *Rhodococcus*.

The *antA* gene encodes the anthranilate 1,2-dioxygenase, enzyme needed to obtain catechol from amino benzoate (Bundy et al. 1998). This gene was found in the consortium, but only *Acinetobacter* harboured one copy of the gene. The *nah* cluster (formed by *nahAa*, *nahAb* and *nahAc* genes) encodes for different subunits of the naphthalene 1,2-dioxygenase enzyme (Ensley et al. 1982), involved in the naphthalene and other PAHs catabolic pathway. In the Noblejas community, a copy of gene *nahAc*, as well as one copy of *nahC*, and both were assigned to *Pseudomonas*. CDSs belonging to *nahAa* were found also in *Pseudomonas* and in *Variovorax* (two copies) but also a copy was found in *Achromobacter* and in a non-classified *Brucellaceae*.

Other genes encoding relevant ring-hydroxylating enzymes are *etbAa* (ethylbenzene) (Iwasaki et al. 2007), *nidA* (phenanthrene) (Sho et al. 2004) and *tphA2* (terephthalate) (Sasoh et al. 2006). *poxA* is a gene encoding a phenol hydroxylase, involved in the degradation of phenol (Kim et al. 1996). Only one copy belonging to *Variovorax* was found in the Noblejas consortium. *etbAa* encodes the alpha subunit of ethylbenzene dioxygenase, an enzyme involved in one of the first step in ethylbenzene degradation. In the Noblejas community one copy of this gene was found *Acrhomobacter*, *Sphingopyxis* and in unclassified *Sphingomonadaceae*. Phenanthrene dioxygenase is encoded by the *nidA* gene. It is an important enzyme starting the ring hydroxylation of phenanthrene. It was found with two copies belonging to *Pseudomonas*. The terephthalate 1,2-dioxygenase enzyme was in the Noblejas consortium with a single copy assigned to *Variovorax*. Other genes identified in the Noblejas consortium were *phdF*, *pht3* and *phtAa* genes. In particular, the *phdF* gene is part of the cluster *phdABCDEF* and is responsible of the cleaveage of 3,4-dyhydroxyphenantrene (Saito et al. 1999) and was found as a single copy in *Novosphingobium* and *Rhodococcus*. The *pht3* and *phtAa* genes encode for enzymes involved in phthalate degradation. More specifically, phthalate 4,5-dioxygenase (Anokhina et al. 2020) and the alpha subunit of phthalate 3,4-dioxygenase (Hu et al. 2021), respectively. Two of the four *pht3* genes copies correspond to *Variovorax* and one to *Novosphingobium* and to an unidentified *Burkholderiaceae* family. Only one copy of *phtAa* was found and it belonged to *Pseudomonas*.

Enzymes encoded by *cmtAb* and *cmtC* genes belong to the p-cumate degradation pathway (Sun et al. 2021). Indeed, the *cmtAb* gene encodes for large terminal subunit of p-cumate dioxygenase, while the *cmtC* gene encodes for a 2,3-dihydroxy-p-cumate 3,4-dioxygenase (Sun et al. 2021). In the Noblejas community both genes were detected. Copies of *cmtAb* were assigned to *Variovorax*, *Xenophilus* and unclassified *Comamonadaceae* and *Rhodospirillales*. Copies of *cmtC* were distributed among *Acrhomobacter* (two copies) and in *Acinetobacter*, *Bosea*, *Xenophilus* and in unclassified *Comamonadaceae*. An unidentified 3-phenylpropionate/trans-cinnamate dioxygenase is the enzyme encoded by the *hcaE* gene (Zampolli et al. 2021). In the Noblejas metagenome, seven copies were found, one corresponding to Novosphingobium four copies to an unidentified *Sphingomonadaceae* and two to *Variovorax*. The *mhpB* gene encodes for the 2,3-dihydro-xyphenylpropionate 1,2-dioxygenase enzyme (Xu and Zhou 2020). In the Noblejas consortium the four copies detected corresponded to *Variovorax* with two copies and *Delftia*, *Rhodococcus* with a single copy each.

Genes involved in central pathways encode enzymes that process the intermediates of peripheral pathways and are highly abundant in the Noblejas consortium. Catechol and protocatechuate are the major intermediaries for aerobic degradation of aromatic compounds. The *catA* and *catE* genes are involved in degradation of cathecol through two different pathways. Catechol 1,2-dioxygenase (ortho pathway) (Neidle and Ornston 1986) is encoded by the *catA* gene while catechol 2,3-dioxygenase (meta pathway) is encoded by the *catE* gene (Williams and Murray 1974). In the Noblejas consortium metagenome, fifteen copies of *catA* and eleven copies of *catE* were found. The copies of *catA* are distributed between *Pseudomonas* (five copies), *Achromobacter* (three copies), *Novospingobium*, *Delftia*, *Acinetobacter*, *Rhodococcus*, *Agrobacterium*, *Bosea* and an unknown genus classified as *Hyphomicrobiales*. The twelve copies of *catE* are distributed between *Agrobacterium*, *Enterobacter*, *Pseudomonas*, *Ochrobactrum*, *Variovorax*, *Bosea*, and two unidentified *Rhizobiaceae* and *Burkholderiaceae*. This finding indicates that most of the major genera present in the consortium can degrade catechol to intermediates of central metabolism. Similarly, many genes implicated in protocatechuate degradation are also present and widely represented in the consortium. For protocatechuate 3,4 dioxygenase (ortho pathway) (Ornston 1966) encoded by the *pcaG* gene, fifteen copies were detected, and were assigned to *Bosea*, *Pseudomonas*, *Achromobacter*, *Variovorax*, *Acinetobacter*, *Rhodococcus*, *Ochrobactrum* and two unidentified *Brucellaceae* and *Rhizobiaceae*. Other gene involved in protocatechuate metabolism is *ligA* gene encoding for protocatechuate 4,5-dioxygenase (meta pathway) (Noda et al. 1990). Five copies were assigned to *Delftia*, an unknow *Burkholderiaceae* and *Sphingobium*.

Homogentisate 1,2- dioxygenase is the enzyme encoded by the *hmgA* gene (Milcamps and de Bruijn 1999). In the Noblejas consortium, the detected twenty-four copies of the gene were harboured by *Variovorax* (six copies), *Pseudomonas* (four copies), *Achromobacter*, *Sphingomonadaceae* (two copies) *Rhodospirillaceae*, *Stenotrophomonas*, *Luiteibacter*, *Chryseobacterium*, *Rhodococcus*, *Novosphingobium*, *Sphingobacterium*, *Caulobacter* and two unidentified *Caulobacteraceae* and *Burkholderiaceae*. The gene *chqB* encodes hydroxyquinol 1,2- dioxygenase enzyme (Daubaras et al. 1996). In the Noblejas metagenome, seven copies of the gene were assigned to *Variovorax*, *Acinetobacter*, *Bosea* and *Pseudomonas*.

Benzoate mineralization via the benzoyl-CoA pathway requires the ligation of acetyl-CoA to benzoate by a benzoate-CoA ligase that is encoded by the *badA* gene (Rather et al. 2010). Nine copies of this gene were identified in the Noblejas consortium corresponding to *Achromobacter*, *Burkholderiaceae* family and *Delftia*. Benzoate is further epoxidated by benzoyl-CoA 2,3 epoxidase, encoded by *boxABCD* gene cluster (Rather et al. 2010). The *boxA* and *boxB* genes encode respectively alpha and beta subunit of benzoyl-CoA 2,3-epoxidase, boxC encodes benzoyl-CoA-dihydrodiol lyase and *boxD* 3,4-dehydroadipyl-CoA semialdehyde dehydrogenase. The whole cluster was found in Noblejas, with eight copies of *boxA* (*Achromobacter*, *Bulkholderiaceae*, *Delftia* and *Variovorax*), five copies of *boxB* (*Bulkholderiaceae* and *Achromobacter*), six copies of *boxC* (*Achromobacter*, *Delftia* and *Bulkholderiaceae*) and three copies of *boxD* (*Variovorax*) were found.

### Metatranscriptomic analysis of the consortium

In order to determine the hydrocarbon degradation activity of the consortium, an RNA-Seq analysis of the consortium growing for 48 h at 28 °C with Diesel as the sole source of carbon and energy was performed.

**Fig. 3:**
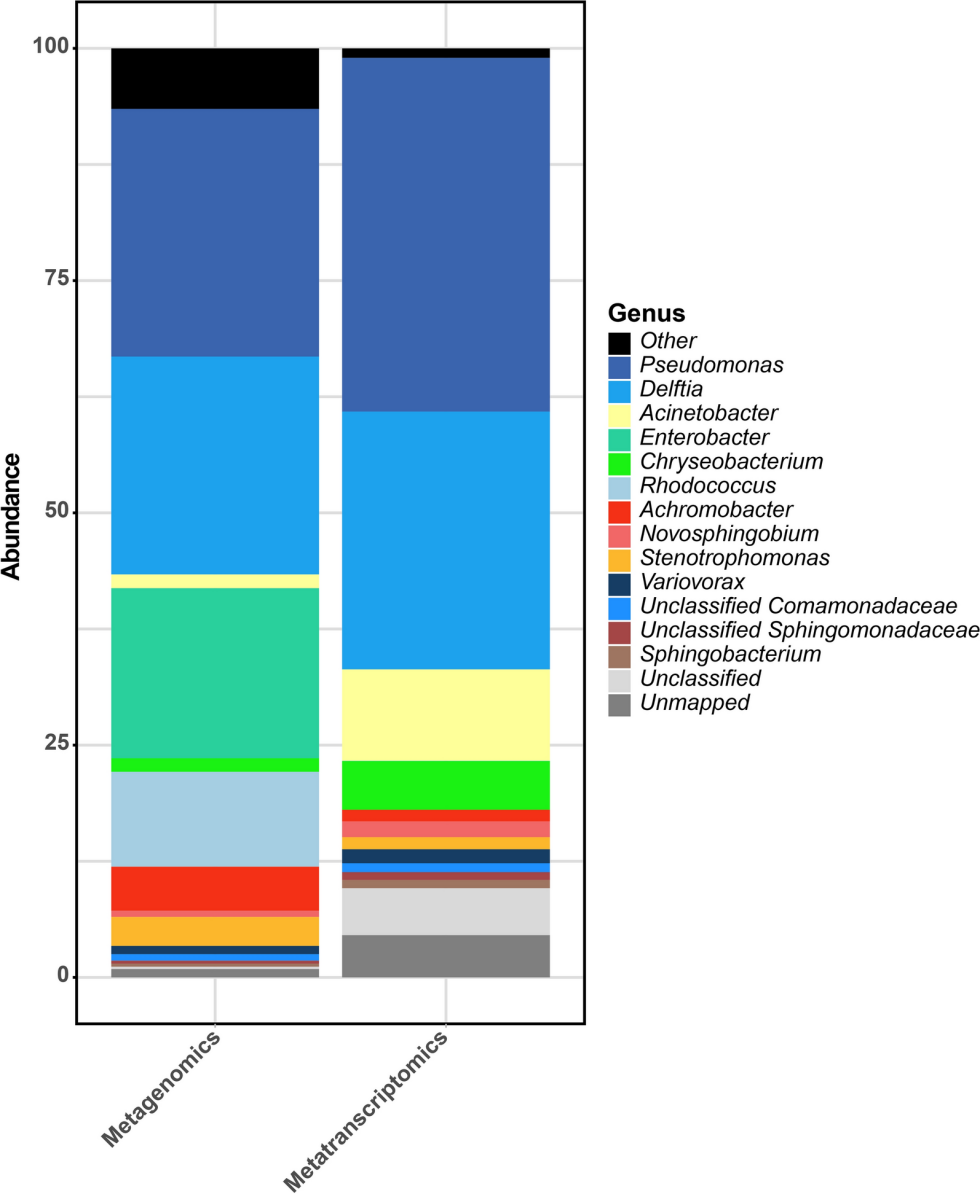
Taxonomic distribution based on RNA-Seq analysis of transcripts from the different genera presents in the metagenomics and metatranscriptomic analysis of Noblejas consortium. Unmapped refers to the proportion of reads that could not be assigned to any contig. Unclassified points to those contigs that could not be assigned to any taxa

Phylogenetic analysis of the obtained transcripts showed that in these conditions the most active populations, in terms of percentage of the transcripts were *Pseudomonas*, *Acinetobacter* and *Delftia* that accounted for more than 75% of the total transcripts. Transcripts from the genera *Novosphingobium*, *Chryseobacterium*, *Sphingobacterium, Variovorax* and *Achromobacter* were also detected (Fig. 3).

Comparing reads from the metagenome with reads from the metatranscriptome, it can be seen that there is a good correspondence between the most abundant populations and the expressed genes. However, the genus *Enterobacter*, very abundant in the consortium, shows very little expression in the metatranscriptome, indicating low activity under the conditions tested. Conversely, *Acinetobacter* and *Chryseobacterium* present an activity higher than expected, considering its relative abundance.

In order to determine the role of these populations in hydrocarbon biodegradation, we also analyzed the transcripts containing the same 35 genes implicated in aliphatic and aromatic hydrocarbons that were determined to be present in the metagenome.

**Fig. 4:**
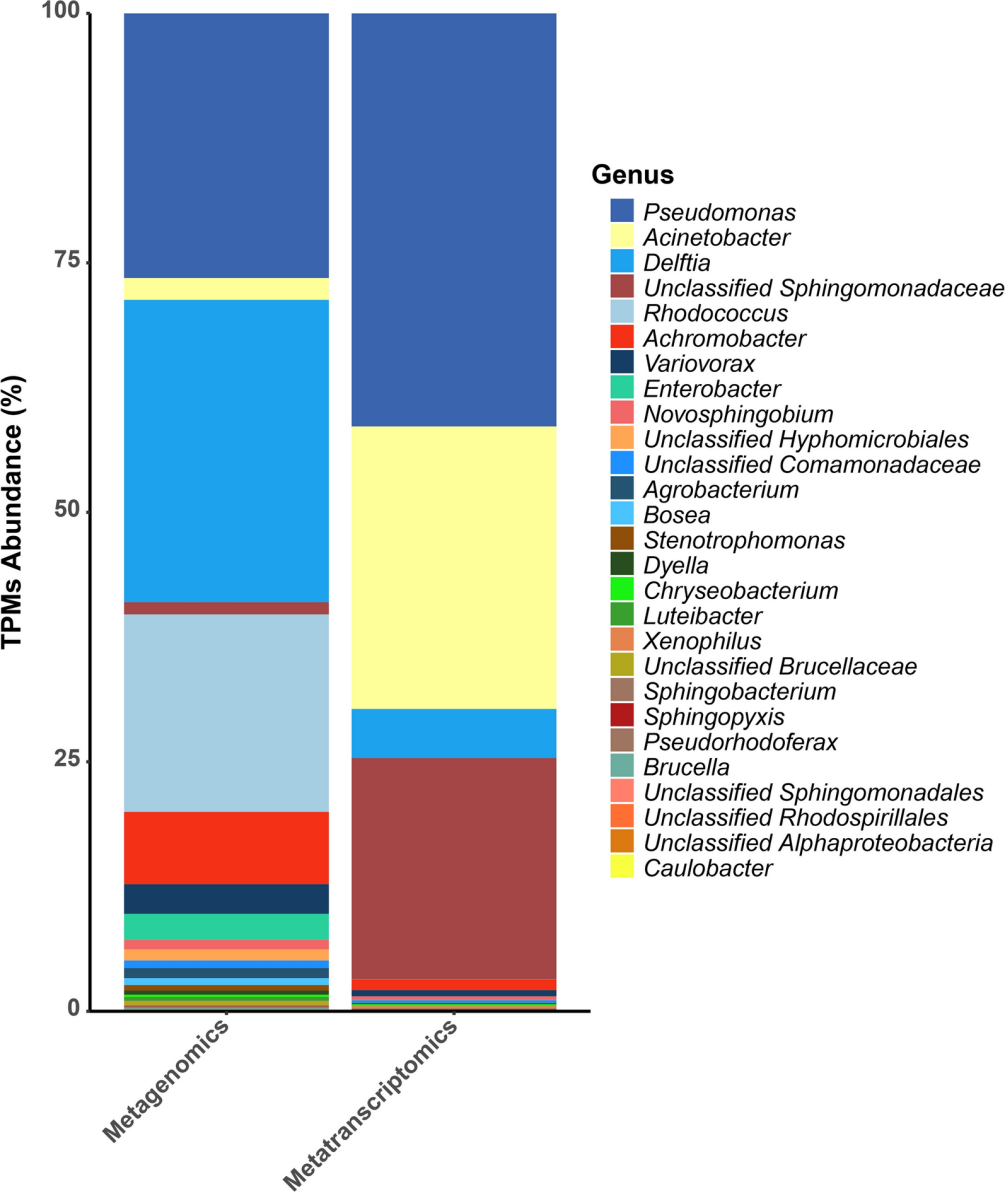
Taxonomic distribution of the reads corresponding to genes implicated in hydrocarbon degradation present in the metagenomic and metatranscriptomics analyses

Regarding taxonomic distribution (Fig. 4), the most active individual populations in hydrocarbon degradation corresponded to *Pseudomonas* (618 TPMs), followed by *Acinetobacter* (454 TPMs), members of the family *Sphingomonadaceae* (356 TPMs) and *Delftia* (79 TPMs). It is interesting to note that bacteria belonging to the *Sphingomonadaceae* family, that show a limited number of genes implicated in hydrocarbon degradation in the metagenome, express a large amount of biodegradation genes, indicating an important participation in hydrocarbon degradation in this consortium, under the tested conditions.

**Fig. 5:**
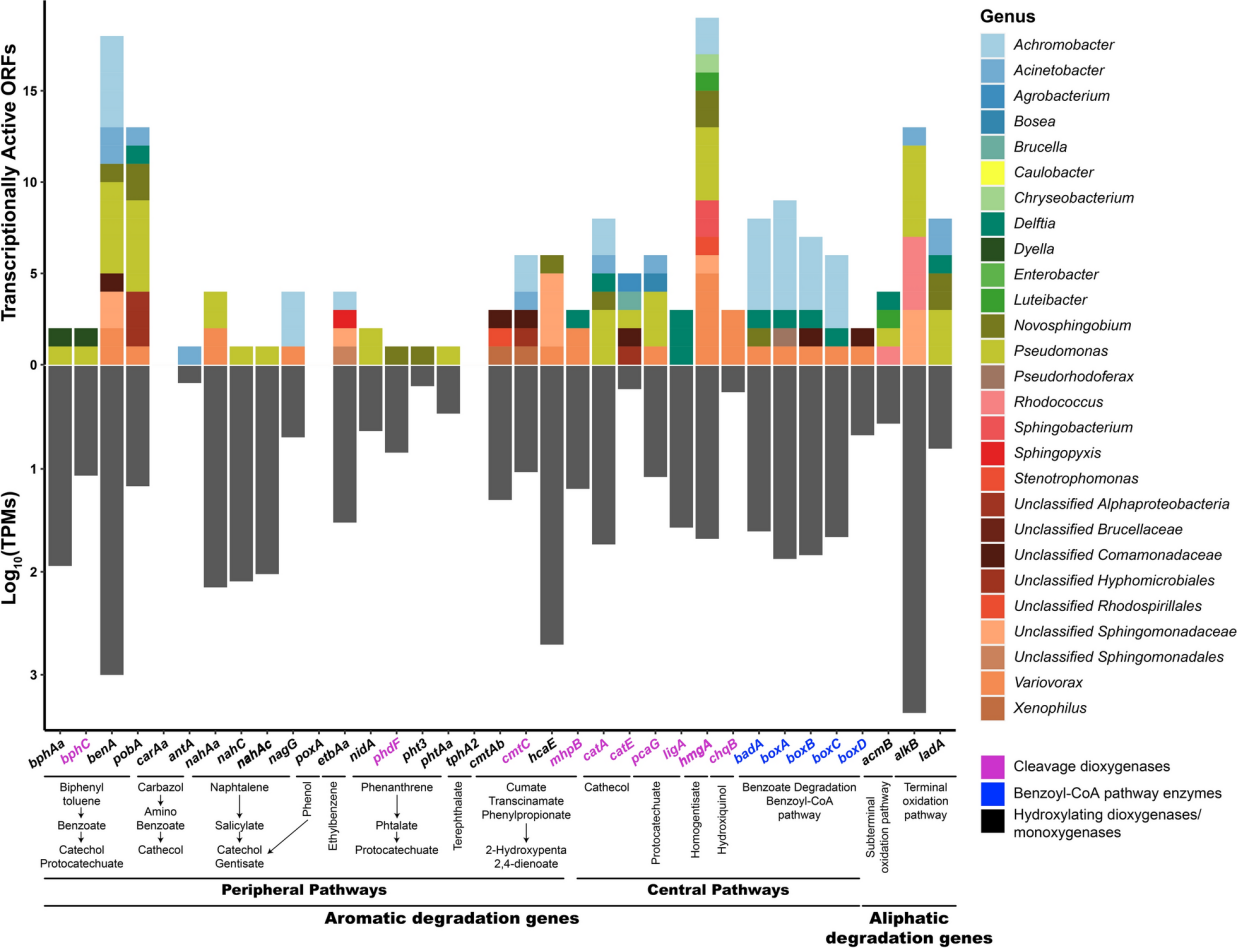
Presence, abundance and and taxonomic assignation at the genus level of the genes encoding enzymes belonging to aromatic and aliphatic degration/metabolism pathways identified in the metatranscriptome and classified to the genus level in the Noblejas Consortium, indicated as a transcriptionally active ORFs. Total TPMs (as Log10) are indicated in the lower part. Abscissa axe labels are grouped according to metabolic pathways and colors indicate enzymatic activity class

Regarding the expression of specific genes, as shown in Fig. 5, in the case of peripheral pathways for aromatic and polyaromatic degradation *Pseudomonas* were again the main actors, expressing genes of the biphenyl and toluene degradation pathways (i.e., *bphAa*, *bphC*, *benA* and *pobA*) while *Achromobacter*, *Novosphingobium*, *Rhodococcus* and *Dyella* showed little or no activity. The degradation of naphthalene initiated by *nahC* and *nahAc* is also carried out by *Pseudomonas* as well as phenanthrene through *phdF*. The coumate degradation carried out by *cmtAb* and *cmtC* appears to be led by individuals of the *Comamonadaceae* family.

In the case of central pathways of aromatic degradation: *Pseudomonas* are expressing genes such as *catE* for catechol degradation (meta pathway), *pcaG* for protocatechuate degradation (ortho pathway) and *hmgA* homogentisate degradation. *Variovorax*, showed some expression for *hmgA* and part of the benzoate and benzoyl-CoA degradation pathway with *boxA* and *boxB*. *Achromobacter* and *Delftia*, on the other hand, showed expression in the entire gene cluster for benzoate and benzoyl-CoA degradation (i.e., *badA*, *boxA*, *boxB* and *boxC*).

For aliphatic hydrocarbons degradation pathway, we have observed expression of genes implicated in terminal (*alkB* and *ladA*) and subterminal oxidation (*acmB*). The most active pathway was the one initiated by *alkB*, produced by *Pseudomonas*, *Acinetobacter* and members of the *Sphingomonadaceae* family. Less expression was found for *acmB* in *Sphingobium*, *Rhodococcus*, *Pseudomonas*, *Novosphingobium* and *Acinetobacter*. Similarly, limited expression of *ladA* genes was observed in *Pseudomonas*, *Novosphingobium*, and *Delftia*.

## Discussion

Bioremediation of complex organic pollutants such as petroleum hydrocarbons often require the synergic activity of different bacterial populations. For this reason, bacterial consortia are developed for bioremediation purposes (Garrido-Sanz et al. 2019, 2022; Martínez-Cuesta et al. 2023; Cao et al. 2022). Here we describe the isolation and characterization of a consortium aimed to degrade total petroleum hydrocarbons based in enrichment culture using diesel as sole carbon and energy source. The Noblejas consortium successful use for bioremediation of polluted soil at the microcosm (200 g) and mesocosm (700 kg) scales has been reported elsewhere (Curiel-Alegre et al. 2024; Curiel-Alegre et al. 2022). The use of the consortium not only reduced by 34% the concentration of hydrocarbons after ninety days, but also increased soil respiration and microbial enzymatic activity at the end of the treatment, indicating a recovery of soil biological activity.

The analysis of the microbiome of the consortium, based on 16S rRNA gene amplicon libraries, has shown ca. 50 ASVs, of which *Pseudomonas*, *Enterobacter*, *Delftia*, *Achromobacter*, *Acinetobacter*, *Novosphingobium*, *Allorhizobium*-*Neorhizobium*-*Rhizobium*, *Ochrobacter* and *Luiteibacter* were the most abundant genera. The analysis of the microbiome based on whole metagenomic DNA analysis showed a similar result and the most abundant genera were the same, indicating that both methods have accurately determined the composition of the bacterial community. Furthermore, from the metagenomic data we have been able to assemble twelve almost complete genomes (MAGs) that were coincident with some of the major populations identified in the consortium.

The bacterial composition of this consortium is similar to the composition of other consortia isolated by enrichment in diesel (Garrido-Sanz et al. 2019; Sulbaran-Bracho et al. 2023; Eze et al. 2021), and contains many populations that have been shown to be efficient in the degradation of hydrocarbons. Many studies report the isolation of *Pseudomonas* strains capable to degrade hydrocarbons. Chebbi et al. 2017 (Chebbi et al. 2017), isolated a strain of *Pseudomonas aeruginosa* (W10) that was able to degrade PAHs from a soil contaminated with used motor oil-contaminated soil, after enrichment on phenanthrene. Strain W10 exhibited the ability to grow on a ample range of hydrocarbons including aliphatics, monoaromatics and polyaromatics. Medić et al. (2020), showed that another strain of *Pseudomonas aeruginosa* had a high capacity for degradation of n-alkanes (n-C16, n-C19) and PAHs (fluoranthene, phenanthrene and pyrene). The degradation efficiency of individual hydrocarbons (initial concentrations of 20 mg/L) ranged between 41 and 98%, depending in the hydrocarbon, over a period of seven days. Other pseudomonads have been described as hydrocarbon degraders. A strain of *Pseudomonas plecoglossicida* can utilize benzene, toluene, and ethylbenzene as primary substrates while co-metabolizing other coexisting contaminants, even at a low temperature (Li et al. 2017)). *Pseudomonas geniculata* is an hydrocarbonclastic bacterium capable to forming biofilm on the fungal hyphae of *Penicillium* sp. with which can degradate hydrocarbons (Utami et al. 2019). A psychrotrophic *Pseudomonas frederiksbergensis* has the ability to degrade aromatic hydrocarbons at temperatures as low as 4 °C. This strain specifically consumed toluene, ethylbenzene, n-propylbenzene and methyl ethyl benzene efficiently (Ruiz et al. 2021).

Another proved hydrocarbon biodegraders highly represented in the Noblejas consortium are bacteria from the genus *Acinetobacter*. Czarny et al. (2020) (Czarny et al. 2020) showed that *Acinetobacter* spp. were increased in presence of diesel oil, heavy metals and PAH. Strains belonging to this genus have been shown to efficiently degrade monoaromatic compounds (Hamme et al. 2003) and PAHs such as naphthalene (Thangaraj et al. 2008), acenaphthene and acenaphthylene (Ghosal et al. 2013, 2016). Furthermore, *Acinetobacter calcoaceticus* can survive and grow in minimal medium with diesel as the only source of carbon. *A. calcoaceticus* was able to degrade 82–92% of aliphatic alkane hydrocarbons (C12-C18) in 28 days (Ho et al. 2020).

An *Achromobacter* species (strain AC15) isolated from mangrove soil was identified as a potent producer of biosurfactant. This surfactant allowed strain AC15 to use high concentrations of pyrene as the sole carbon and energy sources. Results showed that AC15 degraded about 40% of the pyrene, present at an initial concentration of 300 mg/L and reduced the surface tension of the culture medium after 14 d of incubation. AC15 used its emulsification property on the culture medium to improve the solubility of the pyrene in water (Li et al. 2020). *Achromobacter* is one of the important populations in the Noblejas consortium.

*Stenotrophomonas* can also use hydrocarbons as the sole source of carbon and energy. In fact, *Stenotrophomonas* sp. isolated from crude oil-contaminated soil, was able to grow on five alternative PAHs (biphenyl, anthraquinone, phenanthrene, naphthalene, and phenanthridine) as the sole carbon source. The complete genome of this strain revealed that it possesses 145 genes that are involved in the degradation of PAHs.

Other two relevant genera present in the Noblejas Consortium have also been involved in hydrocarbons degradation: *Rhodococcus* and *Delftia*. *Rhodococcus* sp. strain P14 can degrade high-molecular-weight PAHs (three to five rings polycyclic aromatic hydrocarbons including phenanthrene, pyrene, and benzo[a]pyrene) as well as aliphatic hydrocarbons. This strain was also able to degrade aliphatic hydrocarbons (Song et al. 2011). *Rhodococcus jialingiae* is known to be a hydrocarbon-oxidizing bacteria and it has been used in a consortium to successfully biodegrade petroleum (Biktasheva et al. 2018). Garrido-Sanz et al. (2020) isolated *Rhodococcus* sp. WAY2 from a biphenyl degrading consortium (Garrido-Sanz et al. 2018) and showed its ability to degrade multiple polyaromatic compounds. A comparative genomics analysis of this genus showed that hydrocarbon degradation traits were widely distributed among *Rhodococci* (Garrido-Sanz et al. 2020). Regarding *Delftia*, several studies show their ability to degrade hydrocarbons. Lenchi et al. (2020) isolated *Delftia* sp. NL1 from an Algerian oilfield sample. This strain exhibited the ability to degrade more than 66.76% of diesel oil within only 7 days of incubation. *Delftia acidovorans*, is known as a phenanthrene degrading soil bacterium. Shetty et al. (2015) isolated the strain Cs1-4 from PAH contaminated soil in Wisconsin by using phenanthrene as sole carbon source and the complete genome analysis revealed the presence of phenanthrene catabolism genes, styrene degradation via phenyl acetate pathway genes and benzoate degradation via benzoyl-CoA pathway genes. Therefore, the major populations present in the consortium are bacteria known for their ability to degrade hydrocarbons.

We have also used metagenomic data to determine the presence of genes encoding enzymes implicated in hydrocarbon biodegradation. Figure 2 shows a summary of aliphatic and aromatic compounds biodegradation pathways in the most abundant genera present in the Noblejas metagenome. In total, 248 copies of genes implicated in hydrocarbon degradation pathways were found (55 involved in aliphatic degradation and 193 in aromatic catabolism). The Fig. 2 shows the relative abundance of hydrocarbon degradation genes classified by pathway and by genera. It can be observed that all the analyzed pathways are present within the consortium, indicating its suitability for the degradation of complex mixtures of hydrocarbons. Regarding aliphatic hydrocarbons degradation, both terminal and subterminal pathways are represented within the consortium. It can also be inferred that two of the most abundant genera, *Pseudomonas* and *Rhodococcus* have the higher potential for aliphatic hydrocarbons. The higher versatility for hydrocarbon degradation corresponds to *Pseudomonas*, *Delftia*, *Rhodococcus*, *Achromobacter*, *Acinetobacter* and *Variovorax*. These populations, except in the case of *Variovorax*, are among the most abundant in the consortium and have been previously described as hydrocarbonclastic.

**Fig. 6:**
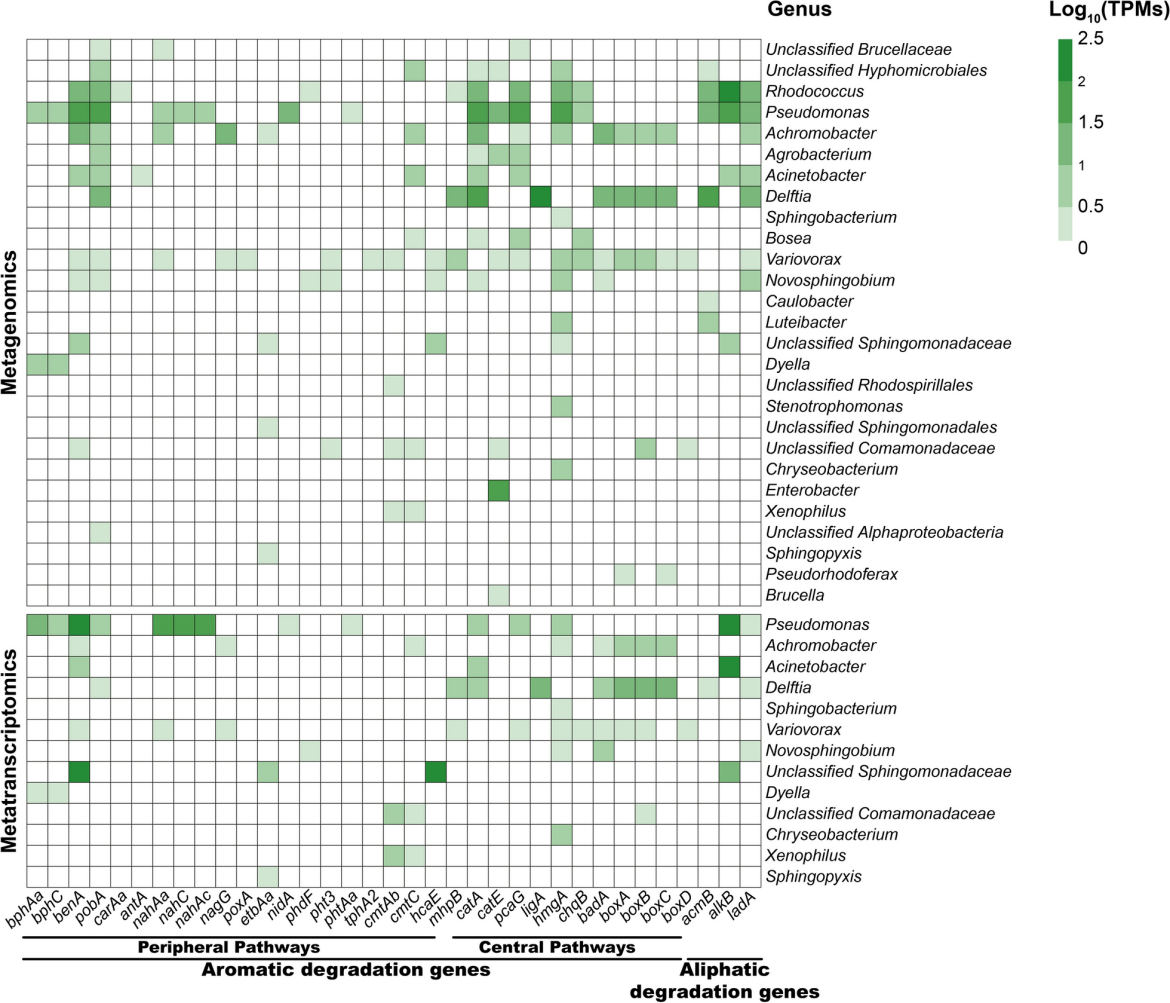
Noblejas Log10(TPMs) heatmap per taxon and gene for the activities studied both by metagenomics and metatranscriptomics approaches

Metatranscriptomic data have shown that the most active populations in the consortium grown using diesel as the sole carbon and energy source were *Pseudomonas*, *Acinetobacter* and *Delftia*, in good agreement with their hydrocarbon degradation potential. Furthermore, the three populations, together with *Sphingobacteria* were shown to be the most active regarding the expression of hydrocarbon degradation genes. It is interesting to note that *Sphingobacteriaceae* that were not detected as a relevant population in the consortium by metagenomic analysis, has been shown by metatranscriptomic analysis to be an active population in hydrocarbon degradation, especially in aliphatic hydrocarbons degradation. This result highlights the importance of metatranscriptomics in the characterization of the consortium. Figure 6 shows a comparison of the potential for hydrocarbon degradation based in the metagenome with the activity shown by the metatranscriptome. This comparison shows a good agreement between metagenome and metatranscriptome. However, some of the genes/pathways present in the metagenome are not actually expressed, as shown in the transcriptome. However, transcriptome analysis is a snapshot of the presence of short-lived RNA in a particular moment. It is likely that these genes/pathways are expressed at other times. Anyway, the fact that most of the genes encoding the biodegradation pathways and the correspondence between the expression of these genes with the major populations in the consortium, indicates that the Noblejas consortium could be a good inoculant for TPHs degradation, as has been shown by its use at microcosm and mesocosm scales (Curiel-Alegre et al. 2024; Curiel-Alegre et al. 2022).

In conclusion, a bacterial consortium suitable to be used as an inoculant in TPHs degradation technologies for soil pollution that require bioaugmentation, such as Ecopiles and Biopiles has been developed through enrichment cultures from TPHs polluted soil. Metagenomic and metatranscriptomic analysis of the consortium has allowed the identification of the bacterial populations present in the consortium and their specific roles in the degradation of a complex pollutant such as diesel. It has been also possible to determine the most active populations in hydrocarbons biodegradation thanks to the metatranscriptomic approach.

## Data Availability

Raw sequences fastq files are available in NCBI Bioproject PRJNA1064703.
